# Supplementary material for: Interactions between the NLRP3-Dependent IL-1β and the Type I Interferon Pathways in Human Plasmacytoid Dendritic Cells
Source: Int J Mol Sci. 2022 Oct 12;23(20):12154. doi: 10.3390/ijms232012154 (PMC9602791; doi:10.3390/ijms232012154)
Supplement: Supplementary file 1 [file ijms-23-12154-s001.zip › ijms-1930155-supplementary.pdf]

International Journal of
Molecular Sciences

MDPI

Supplementary Material

Interactions between the NLRP3-dependent IL-1β and the type I interferon pathways in human plasmacytoid dendritic cells

Dóra Bencze[1,2], Tünde Fekete[1], Walter Pfliegler[3], Árpád Szöőr[4], Eszter Csoma[5], Antónia Szántó[6], Tünde Tarr[6], Attila Bácsi[1], Lajos Kemény[7], Zoltán Veréb[7], Kitti Pázmándi[1,*]

[1] Department of Immunology, Faculty of Medicine, University of Debrecen, Debrecen, Hungary
[2] Doctoral School of Molecular Cell and Immune Biology, University of Debrecen, Debrecen, Hungary
[3] Department of Molecular Biotechnology and Microbiology, Faculty of Science and Technology, University of Debrecen, Debrecen, Hungary
[4] Department of Biophysics and Cell Biology, Faculty of Medicine, University of Debrecen, Debrecen, Hungary
[5] Department of Medical Microbiology, Faculty of Medicine, University of Debrecen, Debrecen, Hungary
[6] Division of Clinical Immunology, Faculty of Medicine, University of Debrecen, Debrecen, Hungary
[7] Department of Dermatology and Allergology, Regenerative Medicine and Cellular Pharmacology Laboratory, Faculty of Medicine, University of Szeged, Szeged, Hungary

Supplementary Figure S1

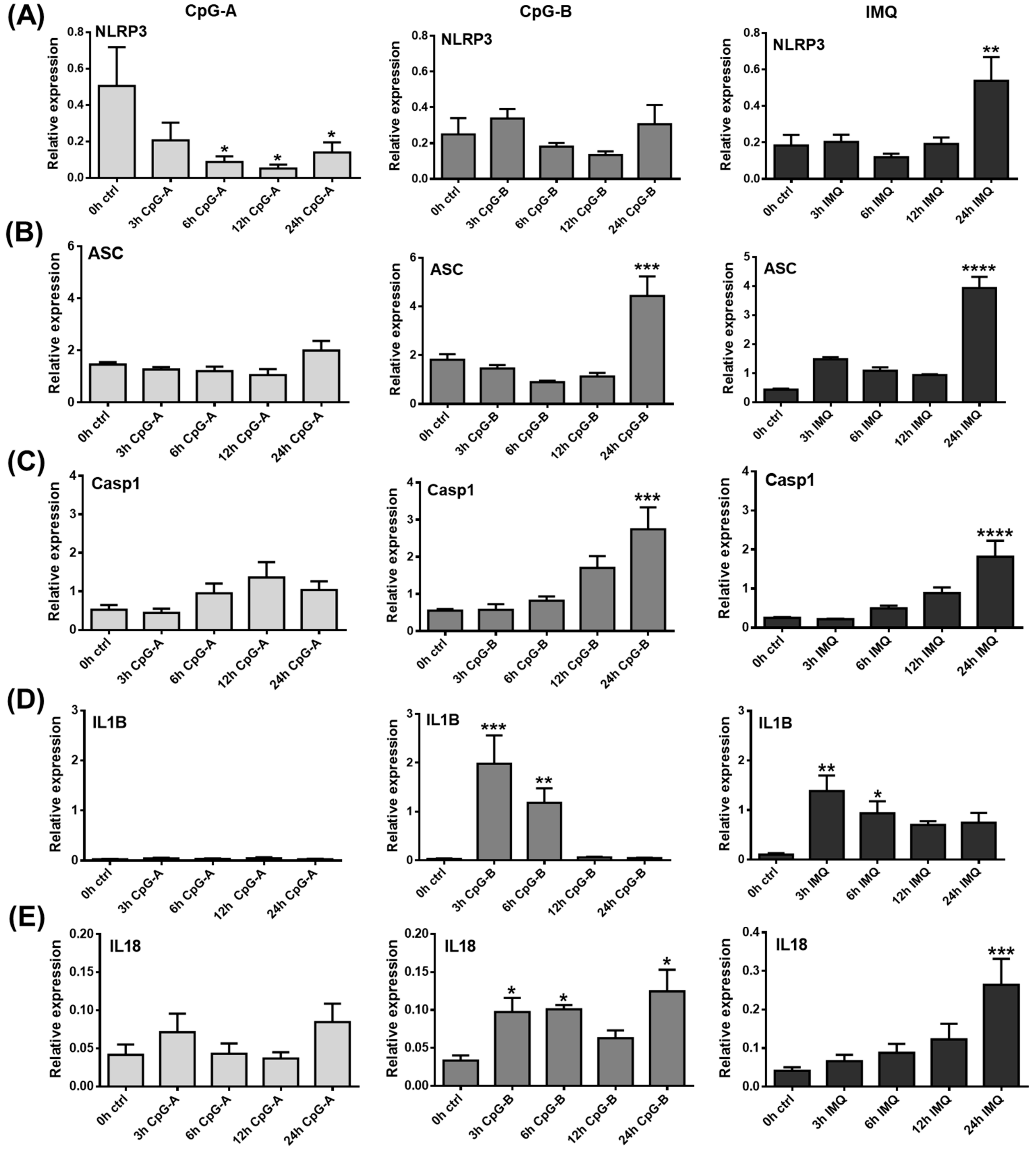

Supplementary Figure S1. Different TLR agonists have various effects on the induction of the NLRP3 inflammasome pathway components at the mRNA level in the human GEN2.2 pDC cell line. GEN2.2 cells were treated with 1 μM CpG-A, 1 μM CpG-B or 5 μg/mL IMQ in a time-dependent manner **(A-E)**. The expression of *NLRP3* **(A)**, *ASC* **(B)**, *Casp-1* **(C)**, *IL1B* **(D)** and *IL18* **(E)** was measured at the mRNA level by qPCR. Data are shown as mean ± SD from 6 independent experiments. Data were analyzed using one-way ANOVA followed by Bonferroni's post-hoc test. *p < 0.05, **p < 0.01, ***p < 0.001, ****p < 0.0001 vs control. ctrl: control, IMQ: imiquimod.

Supplementary Figure S2

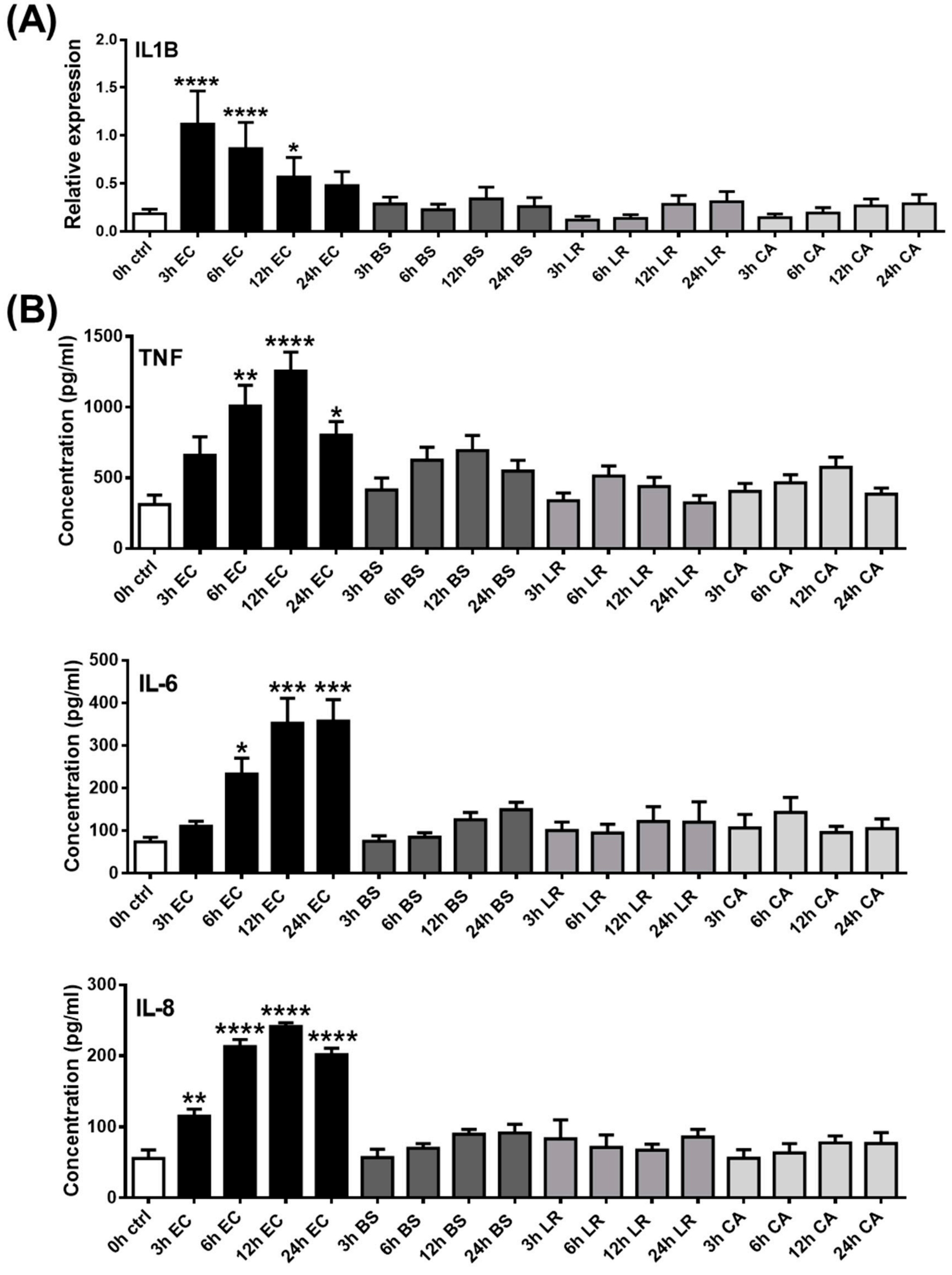

Supplementary Figure S2. Pathogenic bacteria induce the production of pro-inflammatory cytokines in the human GEN2.2 pDC cell line. GEN2.2 cells were treated with *Escherichia coli* (MOI 10), *Bacillus subtilis* (MOI 10), *Lactobacillus rhamnosus* (MOI 10) and *Candida albicans* (MOI 0.01) in a time-dependent manner **(A-B)**. The relative expression of *IL1B* was determined by qPCR **(A)**, while the concentration of TNF-α, IL-6 and IL-8 **(B)** was detected by ELISA. Data are shown as mean ± SD from 4 independent experiments. Data were analyzed using one-way ANOVA followed by Bonferroni's post-hoc test. *$p < 0.05$, **$p < 0.01$, ***$p < 0.001$, ****$p < 0.0001$ vs control. ctrl: control, EC: *Escherichia coli*, BS: *Bacillus subtilis*, LR: *Lactobacillus rhamnosus*, CA: *Candida albicans*.

Supplementary Figure S3

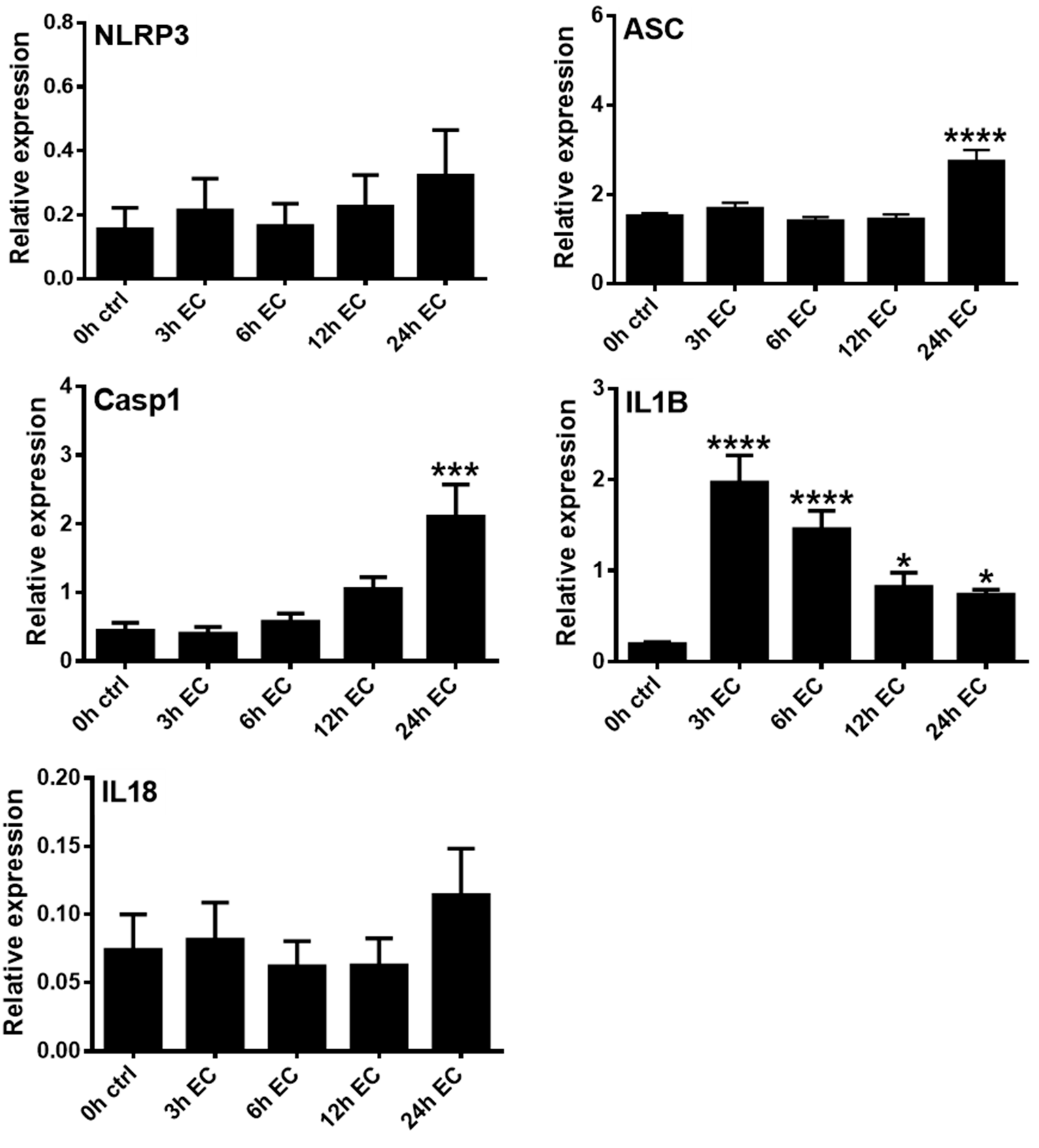

Supplementary Figure S3. *Escherichia coli* induces the expression of the NLRP3 inflammasome pathway components at the mRNA level in the human GEN2.2 pDC cell line. GEN2.2 cells were treated with *Escherichia coli* (MOI 10) in a time-dependent manner. The relative expression of *NLRP3, ASC, Casp-1, IL1B* and *IL18* was measured at the mRNA level by qPCR. Data are shown as mean ± SD from 4 independent experiments. Data were analyzed using one-way ANOVA followed by Bonferroni's post-hoc test. *p < 0.05, ***p < 0.001, ****p < 0.0001 vs control. ctrl: control, EC: *Escherichia coli.*

Supplementary Figure S4

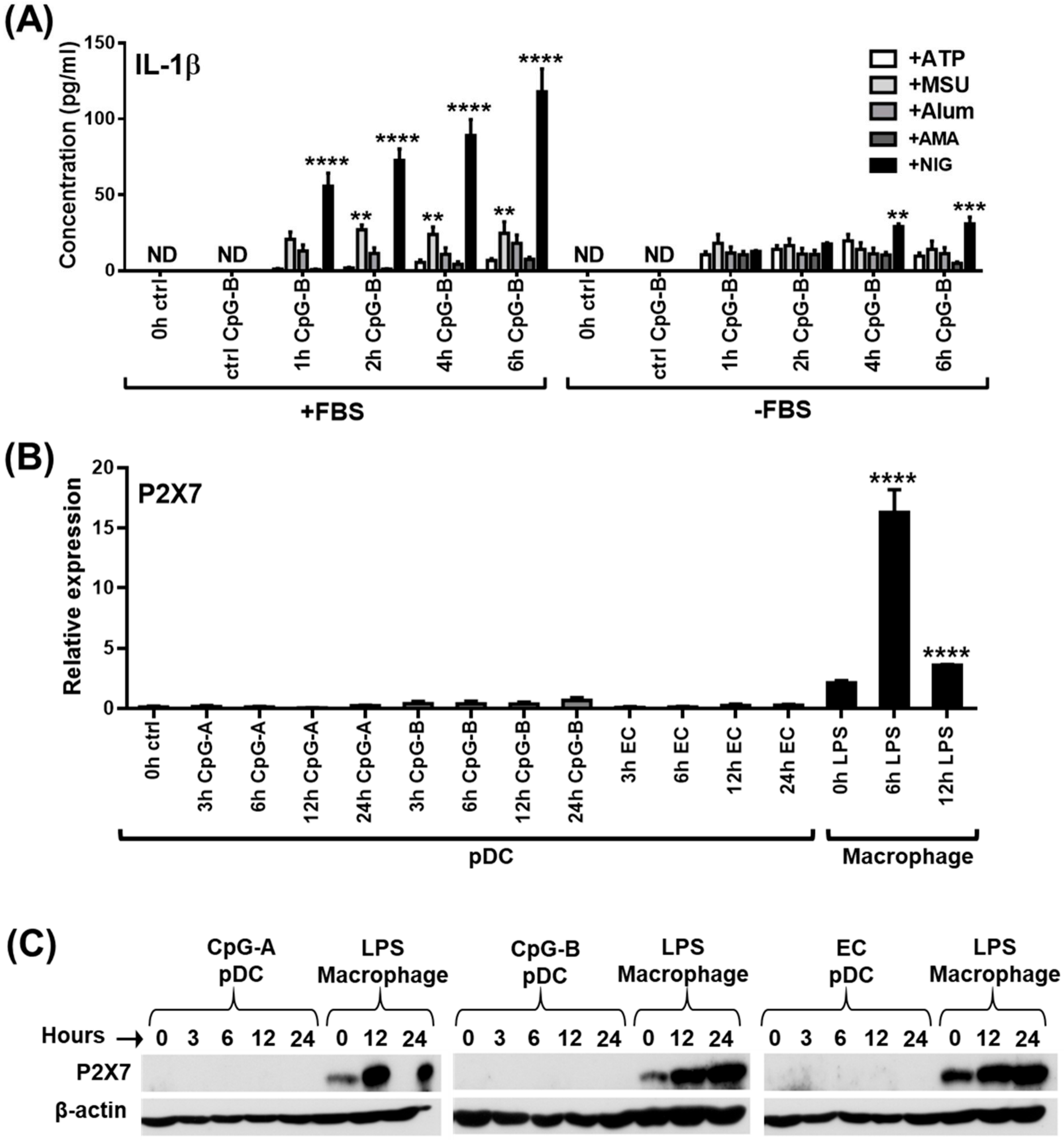

Supplementary Figure S4. Nigericin is the most potent NLRP3 activator in the human GEN2.2 pDC cell line. GEN2.2 cells were stimulated with 1 μM CpG-B for 3 hours in medium with or without FBS. Then, to induce the NLRP3 inflammasome activation cells were incubated in the presence of ATP, MSU, Alum, AMA or nigericin in a time-dependent manner **(A)**. The concentration of IL-1β was measured by ELISA **(A)**. In another experiment, GEN2.2 cells were treated with CpG-A (1 μM), CpG-B (1 μM) or *Escherichia coli* (MOI 10), while primary macrophages were activated with LPS (250 ng/mL) in a time-dependent manner **(B-C)**. The expression of the *P2X7* receptor was determined by qPCR at the mRNA level **(B)**. The representative blots show the protein level of P2X7 measured by western blotting **(C)**. Data are shown as mean ± SD from 4 independent experiments. Data were analyzed using one-way ANOVA followed by Bonferroni's post-hoc test. **$p < 0.01$, ***$p < 0.001$, ****$p < 0.0001$ vs control. ctrl: control, ND: not determined, MSU: uric acid sodium salt, AMA: antimycin A, NIG: nigericin, FBS: fetal bovine serum, EC: *Escherichia coli*.

Supplementary Figure S5

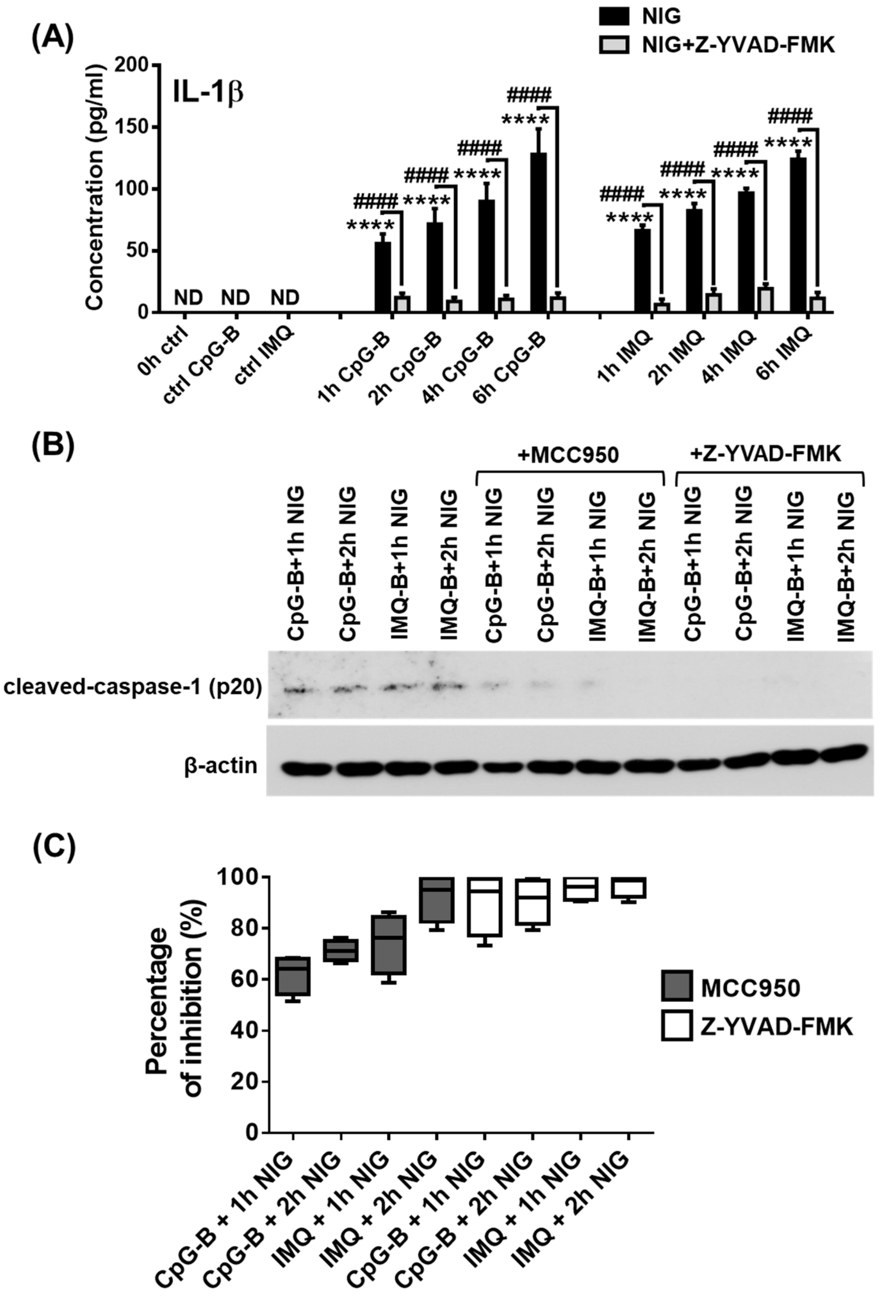

Supplementary Figure S5. The IL-1β secretion of the human GEN2.2 pDC cell line is caspase-1 dependent. GEN2.2 cells were activated with 1 μM CpG-B or 5 μg/mL IMQ for 3 hours, then incubated in the presence of 20 μM nigericin in a time-dependent manner. In parallel experiments, cells were treated with Z-YVAD-FMK (20 μM) for the last 30 minutes of TLR stimulation, prior to nigericin exposure. The concentration of IL-1β was measured by ELISA **(A)**. In other experiments, cells were primed with 1 μM CpG-B or 5 μg/mL IMQ for 3 hours, then incubated with 20 μM nigericin alone or in the presence of 1 μM MCC950 or 20 μM Z-YVAD-FMK for 1 or 2 hours. Cleaved-caspase-1 levels were determined by western blotting as shown in the representative blot **(B)**, and the percentages of the inhibition induced by MCC950 and Z-YVAD-FMK were calculated **(C)**. Data are shown as mean ± SD from 4 independent experiments. Data were analyzed using one-way ANOVA followed by Bonferroni's post-hoc test. ****p < 0.0001 vs control, ####p < 0.0001. ctrl: control, ND: not determined, NIG: nigericin, IMQ: imiquimod.

Supplementary Figure S6

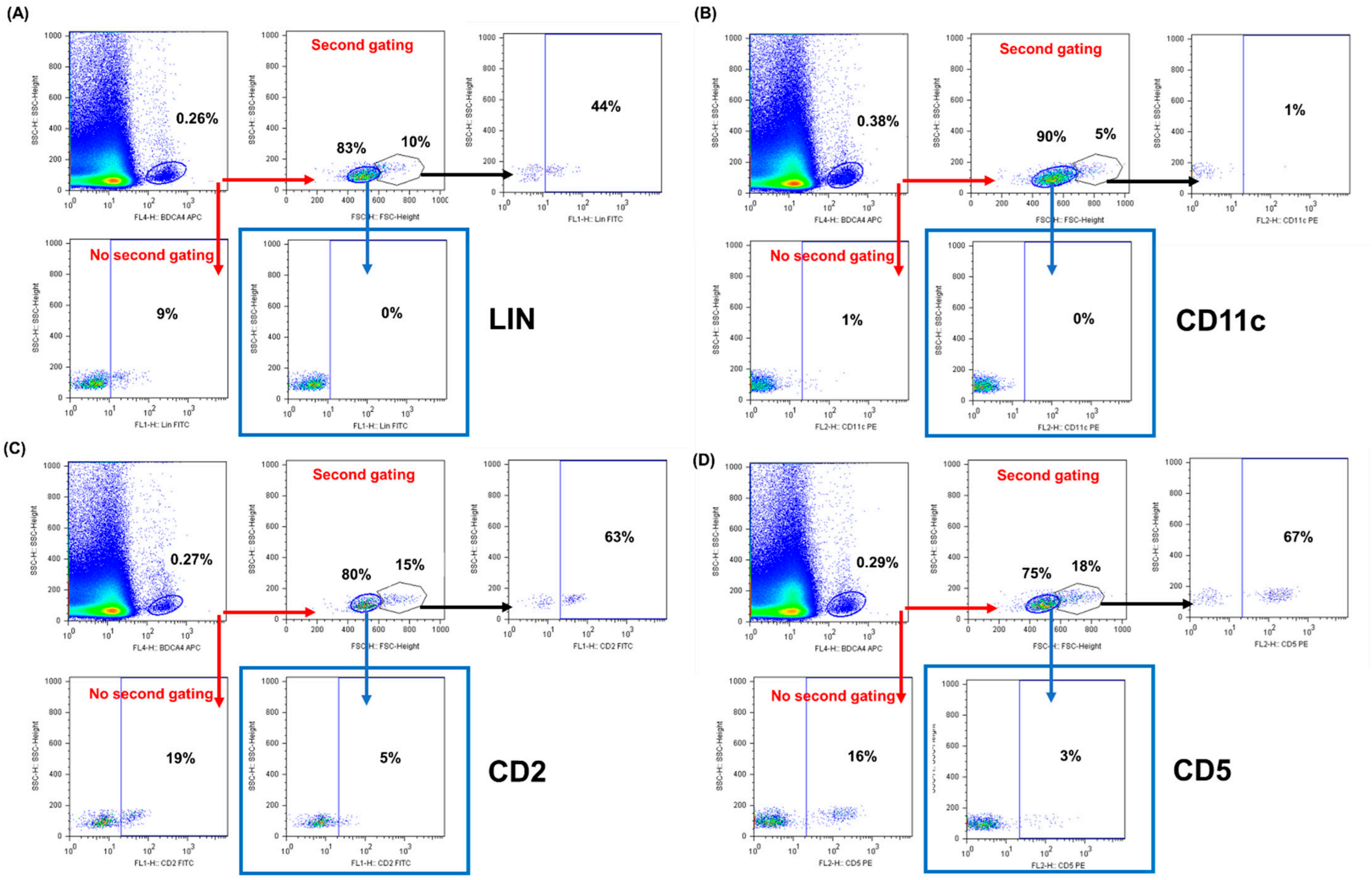

Supplementary Figure S6. Gating strategy of primary human pDCs for flow cytometric analysis I. Representative dot plots show the gating strategy to identify pDCs in PBMCs from healthy volunteers **(A-D)**. First, pDCs were identified as positive for BDCA4 **(A-D)**. After that, the distribution of the cells within the "pDC gate" was analyzed by backgating on the light scatter parameters to exclude non-pDCs. BDCA4+ cells in the region 400-600 of FSC were defined as pDCs **(A-D)**. Percentage of positive cells for LIN **(A)**, CD11c **(B),** CD2 **(C)** and CD5 (**D**) was determined. Eventually, the cell population gated on BDCA4 positivity and light scatter properties (indicated by blue rectangles) was used to analyze the NLRP3 inflammasome pathway components by flow cytometry **(A-D).**

Supplementary Figure S7

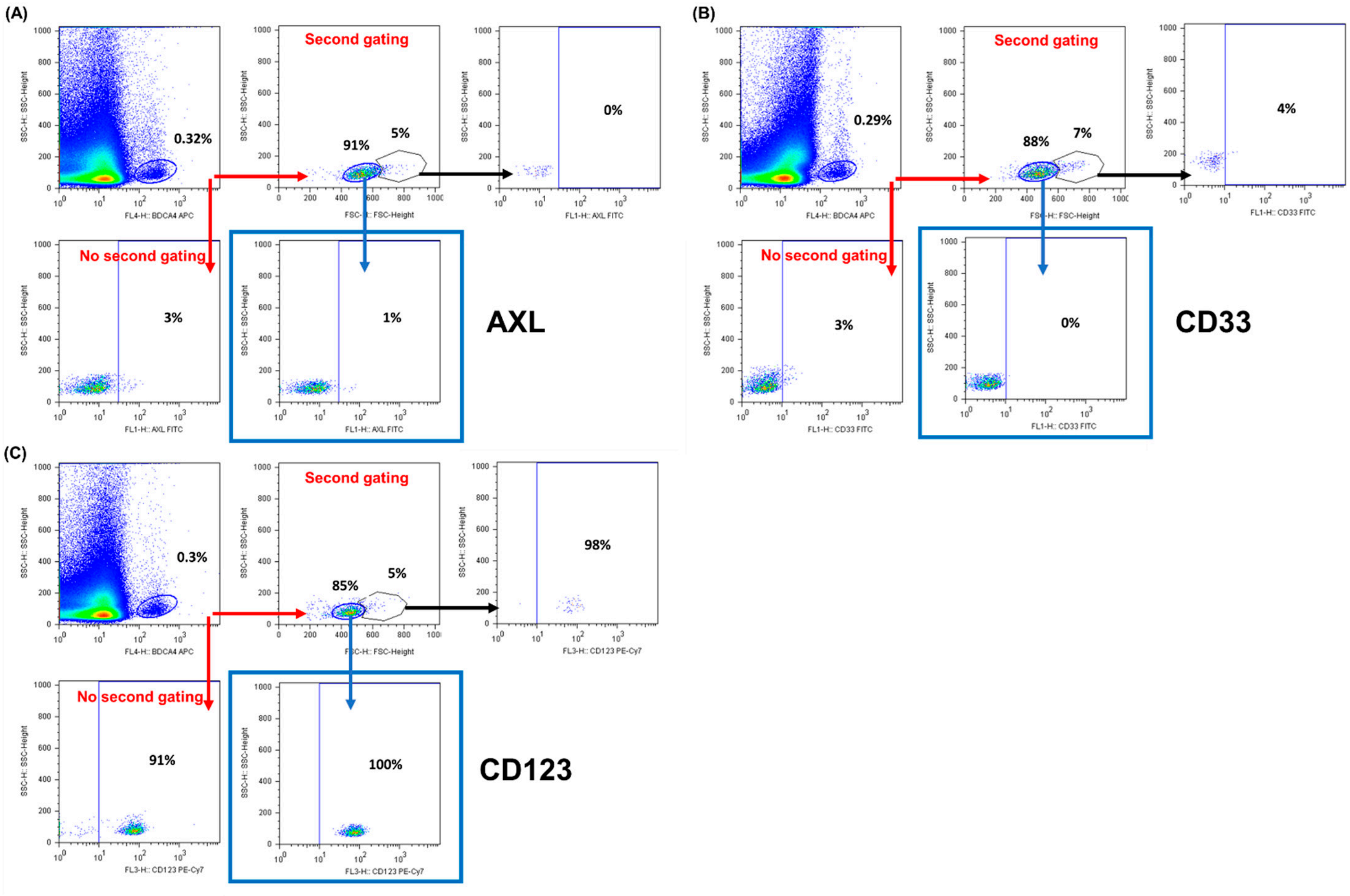

Supplementary Figure S7. Gating strategy of primary human pDCs for flow cytometric analysis II. Representative dot plots show the gating strategy to identify pDCs in PBMCs **(A-C)**. First, pDCs were identified as positive for BDCA4 **(A-C)**. After that, the distribution of the cells within the "pDC gate" was analyzed by backgating on the light scatter parameters to exclude non-pDCs. BDCA4+ cells in the region 400-600 of FSC were defined as pDCs **(A-C)**. Percentage of positive cells for AXL **(A)**, CD33 **(B)** and CD123 **(C)** was investigated. Eventually, the cell population gated on BDCA4 positivity and light scatter properties (indicated by blue rectangles) was used to analyze the NLRP3 inflammasome pathway components by flow cytometry **(A-C)**.

Supplementary Figure S8

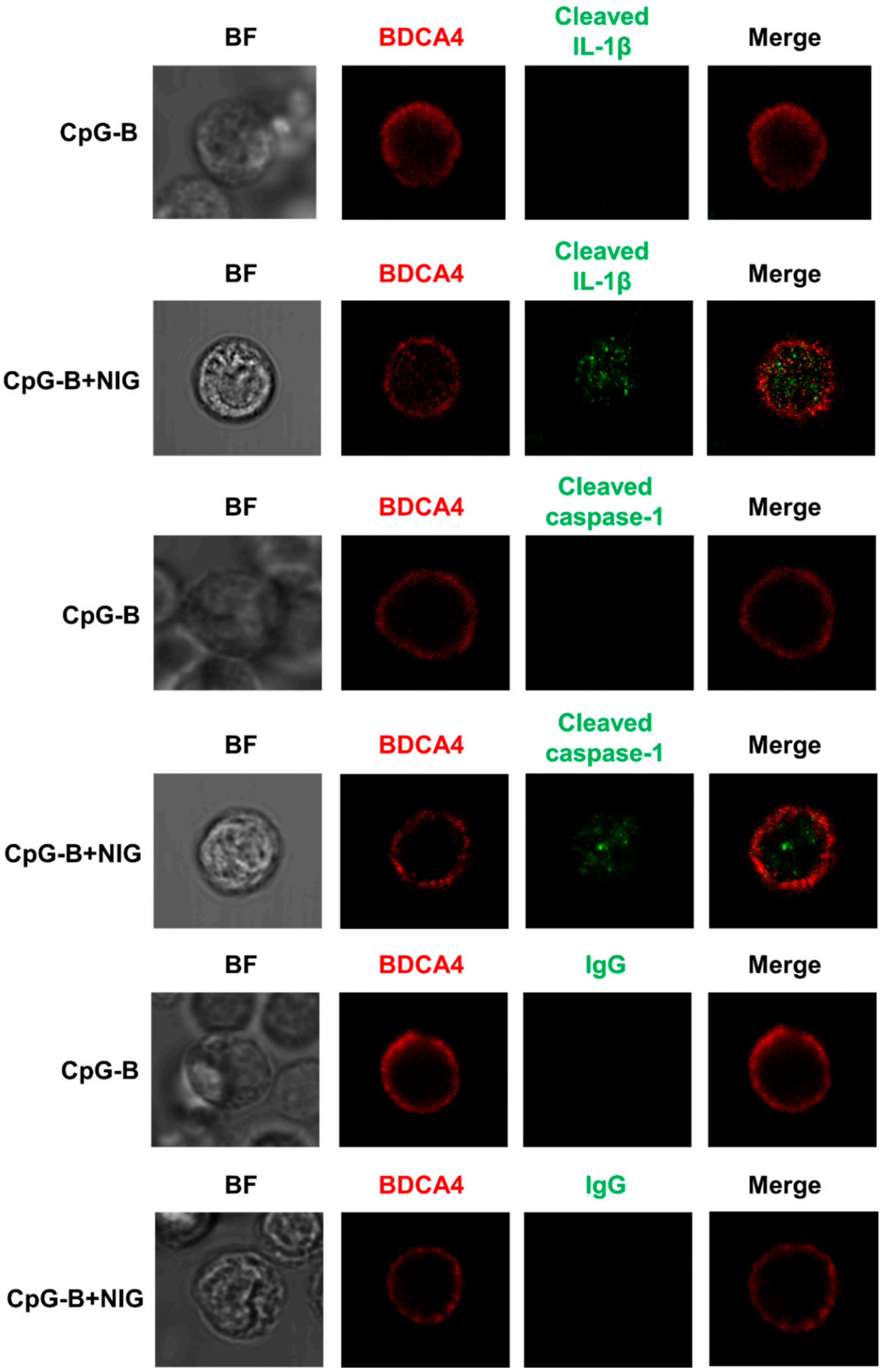

Supplementary Figure S8. Confocal microscopic analysis of the cleaved forms of IL-1β and caspase-1 in primary human pDCs. Peripheral blood samples were taken from healthy volunteers and PBMCs were separated using Ficoll-Paque gradient centrifugation. PBMCs were activated with 1 μM CpG-B for 2.5 hours alone or further incubated in the presence of 20 μM nigericin for 1 hour after priming with CpG-B. Following fluorescent staining pDCs (red) and the cleaved forms of IL-1β and caspase-1 (green) were visualized by confocal laser scanning microscopy. Representative images from 4 independent experiments are shown. BF: bright field, NIG: nigericin.

Supplementary Figure S9

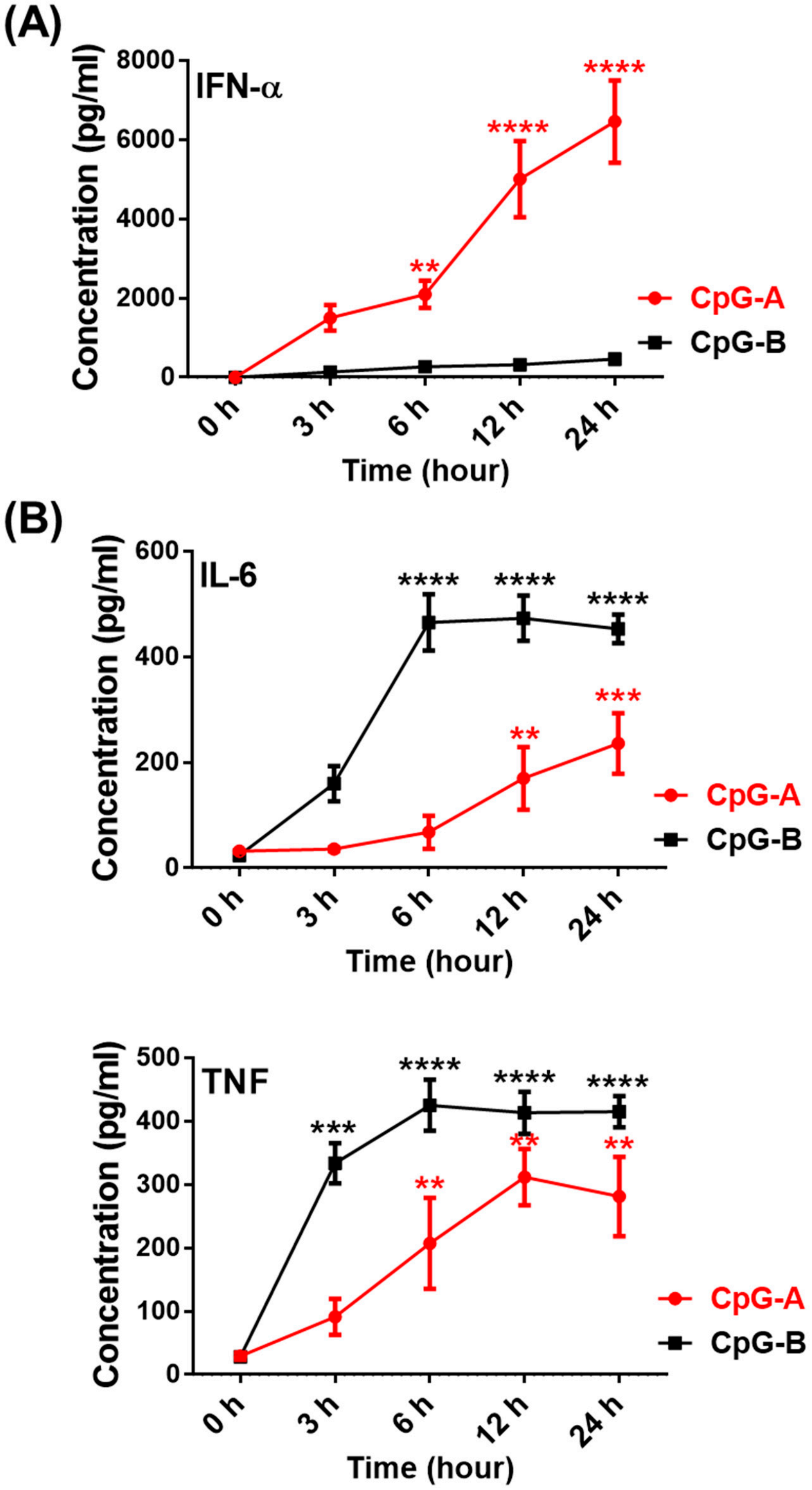

Supplementary Figure S9. The synthetic TLR9 ligands, CpG-A and CpG-B differ in their type I IFN inducing ability. GEN2.2 cells were activated with 1 μM CpG-A or 1 μM CpG-B in a time-dependent manner. IFN-α **(A)**, IL-6 and TNF-α **(B)** concentrations were determined by ELISA. Data are shown as mean ± SD from 4 independent experiments. Data were analyzed using one-way ANOVA followed by Bonferroni's post-hoc test. **$p < 0.01$, ***$p < 0.001$, ****$p < 0.0001$ vs control.

Supplementary Figure S10

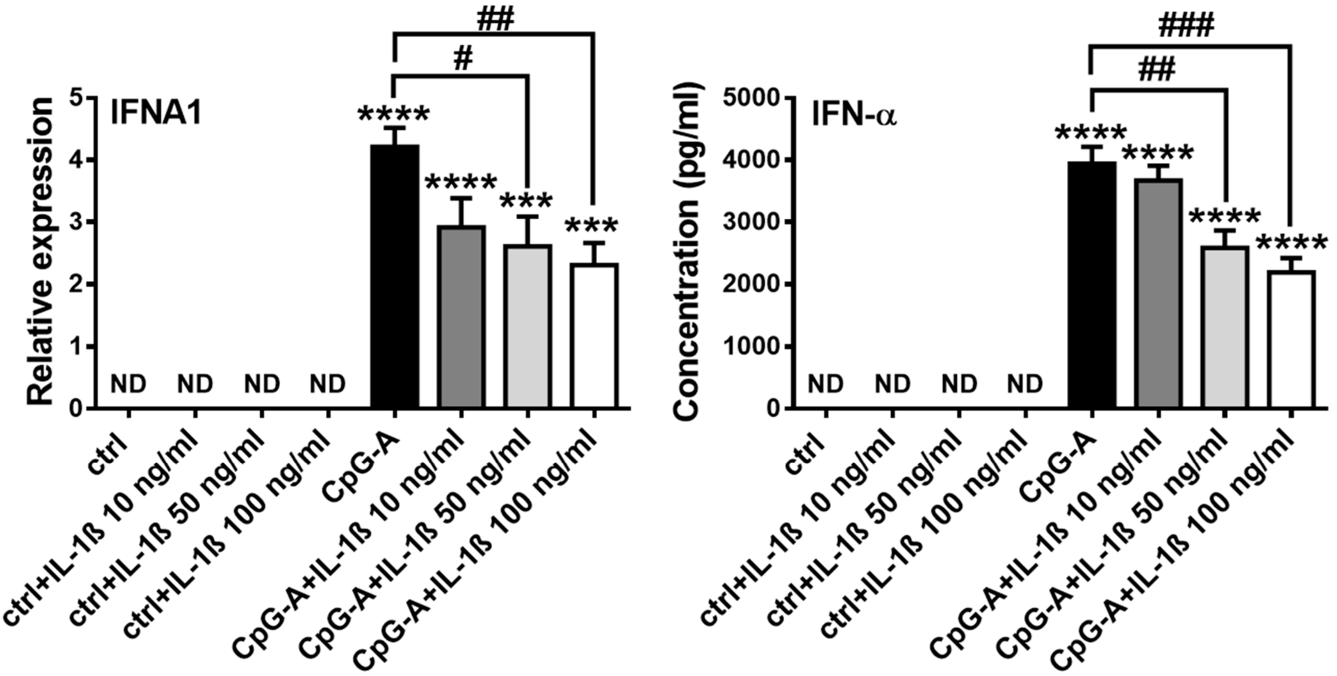

Supplementary Figure S10. IL-1β inhibits IFN-α production in the human GEN2.2 cell line. GEN2.2 cells were pretreated with recombinant human IL-1β for 30 minutes in a concentration dependent manner, then activated with 1 μM CpG-A for 12 hours. The relative expression of *IFNA1* was determined by qPCR, while the concentration of IFN-α was measured by ELISA. Data are shown as mean ± SD from 4 independent experiments. Data were analyzed using one-way ANOVA followed by Bonferroni's post-hoc test. ***$p < 0.001$, ****$p < 0.0001$ vs control, #$p < 0.05$, ## $p < 0.01$, ###$p < 0.001$. ctrl: control, ND: not determined.

Supplementary Figure S11

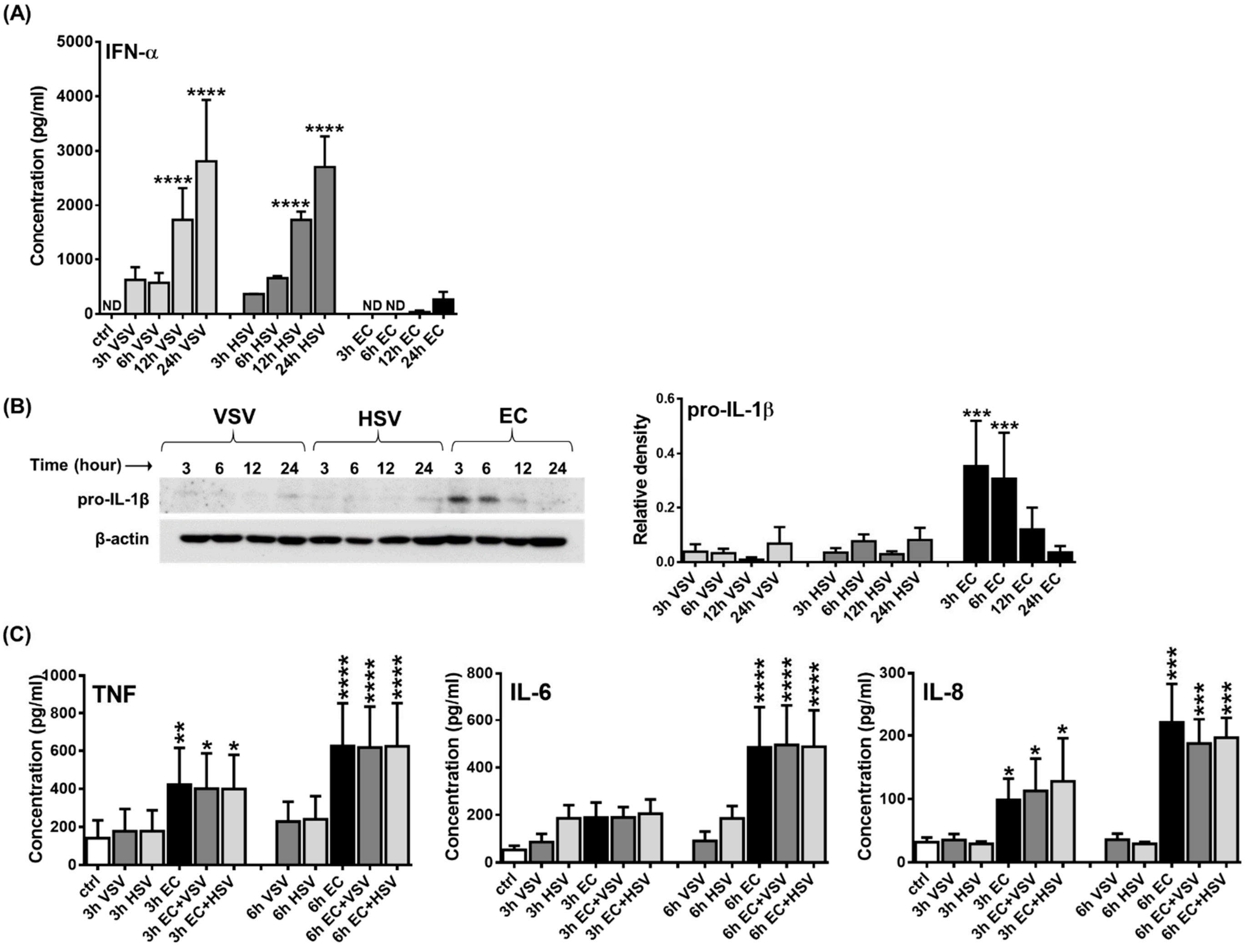

Supplementary Figure S11. Viruses and bacteria differ in their type I IFN and IL-1β inducing ability and the viral pre-treatment does not influence the *Escherichia coli* induced pro-inflammatory cytokine and chemokine secretion in the human GEN2.2 pDC cell line. GEN2.2 cells were activated with VSV, HSV or *Escherichia coli* at a MOI of 10 in a time-dependent manner **(A-B)** or cells were pre-treated with VSV or HSV (MOI 10) for 3 hours, then incubated in the presence of *Escherichia coli* (MOI 10) for an additional 3 or 6 hours **(C)**. The concentration of IFN-α was detected by ELISA **(A)**. The expression of pro-IL-1β was measured by western blotting **(B)**. A representative blot is shown in **(B).** The concentration of TNF-α, IL-6 and IL-8 was also determined by ELISA **(C)**. Data are shown as mean ± SD from 4 independent experiments. Data were analyzed using one-way ANOVA followed by Bonferroni's post-hoc test. *$p < 0.05$, **$p < 0.01$, ***$p < 0.001$, ****$p < 0.0001$ vs control. ctrl: control, ND: not determined, VSV: *Vesicular Stomatitis Virus*, HSV: *Herpes Simplex Virus*, EC: *Escherichia coli.*

Supplementary Figure S12

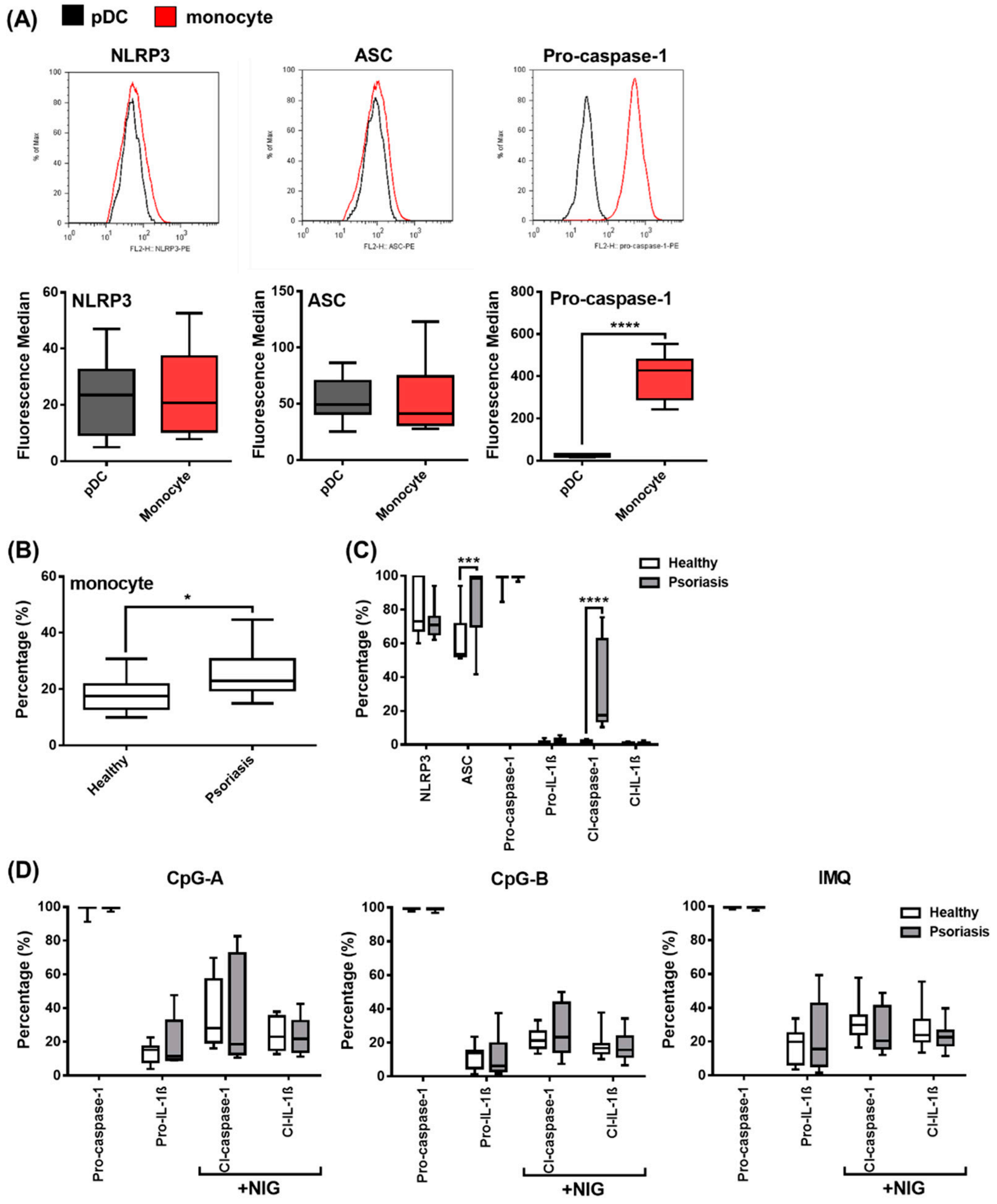

Supplementary Figure S12. Analyzing the expression profile of NLRP3 inflammasome components in pDCs and monocytes, and comparing their distribution in monocytes from healthy volunteers and psoriatic patients. Peripheral blood was taken from healthy volunteers and psoriasis patients and PBMCs were isolated using Ficoll-Paque gradient centrifugation. The pDCs and monocytes from PBMCs were analyzed separately by flow cytometry. The fluorescence intensity of NLRP3, ASC and pro-caspase-1 in the gated pDC and monocyte populations was measured using intracellular flow cytometry **(A)**. Representative histograms are shown in the upper part of the panel **(A)**. In parallel experiments PBMCs were left untreated **(B-C)** or were stimulated with 1 μM CpG-A or 1 μM CpG-B or 5 μg/mL IMQ for 2.5 hours **(D)**. Alternatively, priming was followed by a 1 hour treatment with nigericin (20 μM) to induce the expression of the cleaved forms of IL-1β and caspase-1, **(D).** The percentage of monocytes in PBMCs from psoriasis and healthy subjects is shown **(B)**. The baseline **(C)** and the activation induced **(D)** expression of NLRP3 pathway components in monocytes were determined by intracellular flow cytometry. Bar graphs represent the mean ± SD of 14 independent experiments. Data were analyzed using paired Student *t* test (**A-B**) and two-way ANOVA followed by Bonferroni's post-hoc test (**C-D**). ****p < 0.0001 vs pDC or ***p < 0.001 ****p < 0.0001 vs healthy volunteers. NIG: nigericin, IMQ: imiquimod.
